# Safety Evaluation of Essential Oil Formulation (EOF) in Albino Wistar Rats: An Experimental Study

**DOI:** 10.1155/bmri/8254505

**Published:** 2026-10-17

**Authors:** Ansam Naji Aboud Alhassani

**Affiliations:** ^1^ Department of Pharmacology College of Pharmacy Imam Ja′afar Al-Sadiq University Al-Muthanna Iraq

**Keywords:** acute toxicity, essential oil formulation, linoleic acid, phytochemical analysis, subchronic toxicity

## Abstract

**Background:**

Many people in Iraq utilize olive, rosemary, and lavender oils for cosmetics. However, there is no significant evidence supporting the plant′s safety regarding its oils.

**Objectives:**

This study is aimed at examining the acute and subchronic toxicities of essential oil formulation (EOF) in albino Wistar rats using various parameters.

**Materials and Methods:**

The collected EO was analyzed by GC‐MS. For acute toxicity assay, 18 rats were divided equally into 3 groups of 6 animals each as low‐dose, median‐dose, and high‐dose and received a single oral dose (500, 1000, and 2000 mg/kg, respectively). Whereas for subchronic toxicity assay, a total of 30 rats were allocated to five groups of six experimental rats were randomly assigned to Group I (control), which received tap water, whereas Groups II–V received 50, 150, 250, and 350 mg/kg of EOF prepared from olive, rosemary, and lavender oils, respectively. Rats underwent a 90‐day repeated‐dose oral toxicity investigation in accordance with OECD 408 (Oral Toxicity‐Fixed Dose Procedure). Following a humane euthanasia using sodium pentobarbital, blood samples were collected for liver and renal function tests, hematological parameters, and electrolyte balance indicators. In contrast, the liver and kidney were removed for histopathological analysis.

**Results:**

Olive oil was mostly composed of oleic acid with tiny amounts of linoleic and palmitic acids. The principal components of rosemary oil include *α*‐pinene, 1,8‐cineole, and camphor, whereas lavender oil contains linalool, linalyl acetate, 1,8‐cineole, *β*‐ocimene, terpinen‐4‐ol, and camphor. EOF′s NOAEL exceeded 2000 mg/kg body weight. EOF‐treated rats at 50, 150, 250, and 350 mg/kg exhibited no toxicity or death after 3 months of treatment. Liver and renal function tests, electrolyte balance, and blood marker indicators were not significantly affected in EOF‐treated rats compared with controls. Liver and renal histopathology showed unremarkable changes after treatments with various doses of EOF.

**Conclusions:**

No significant toxicity was observed for assessed parameters, including the immunological, blood, enzyme, and essential organ systems. Thus, EOF is considered relatively safe and nontoxic for usage as its NOAEL exceeded 2000 mg/kg body weight.

## 1. Introduction

Essential oils (EOs) and odorous compounds are soluble in ethanol, but not water, and their chemical makeup includes esters, aldehydes, alcohols, ketones, and terpenes. Since the introduction of gas chromatography‐mass spectrometry (GC‐MS), the list of compounds found at minuscule or trace levels in EOs has grown and continues to grow. Terpenes, alcohols, aldehydes, ketones, and esters are commonly found in EOs [1]. Globally, about 80% of the population relies on herbal medicine [2]. This high demand is mirrored in Iraq, where herbal remedies are deeply rooted in local culture [3]. Different species of herbs and plant parts might have EOs with varied compositions, some of which are extremely hazardous to humans even in small doses. Thus, some EOs might cause mucosal soreness, vomiting, epigastric pain, diarrhea, seizures, kidney failure, hepatotoxicity, dizziness, and depression of the central nervous system [4], especially when not used randomly and without caution. In this regard, terpineol from *Thymus eriocalyx* oil is considered microsomal mutagenic, and linalool in thymus oil can kill skin cells [5]. Consequently, human‐use EO should be studied for toxicity [6].

A wealth of biodiversity is available for lead chemical identification and therapeutic development, as around 70% of the active components currently used or in clinical studies are natural [7]. Drug safety must be considered along with pharmacodynamic and pharmacokinetic factors. Hepatotoxicity and nephrotoxicity are the main reasons clinical trials stop [8]. However, 90% of Middle Easterners use traditional medicine, especially EOs belonging to the families Lamiaceae and Oleaceae [9], whereas aromatic oils have been used for cooking and fumigation [10]. The EOs have risen in popularity over the past decade, particularly those that function in society as holistic integrative modalities alongside traditional medicinal treatments, where many Americans substitute EOs for other prescribed medications [3]. Recently, studies have continued to determine the EOs′ cytotoxic, antimicrobial, anti‐inflammatory, antiplatelet aggregation, anticancer, and antioxidant properties. Although medicinal plants have a high therapeutic index, their natural metabolites can harm humans, animals, and the environment if not used scientifically [9]. Based on recent studies, aromatic plants′ EO derived from olive, rosemary, and lavender are ethnopharmacologically approved with many beneficial effects to several human diseases [11]. Up to date, there is no detailed published data on no‐observed‐adverse‐effect level (NOAEL) in Iraq, which is the highest tested dose of a substance that causes no detectable adverse changes in morphology, growth, development, or lifespan in exposed animals, and it serves as a key point of departure for determining safe exposure levels in chemical and drug risk assessments [12]. Therefore, this study is aimed at examining the acute and subchronic toxicities of these EOs in vivo using hematological, biochemical, and histopathological methods; also, it is aimed at determining the NOAEL of the essential oil formulation (EOF) that was prepared from these extracted oils.

## 2. Materials and Methods

### 2.1. Collecting and Identifying Plants

The fresh olive, rosemary, and lavender leaves were collected from the Tishk International University botanical garden in Erbil, the Kurdistan Region, Iraq. Then, a specialized botanist authenticated the leaves. Voucher Specimen No. EOF/2023 was conserved in the herbarium of the Department of Pharmacognosy at Tishk International University, Erbil, Iraq.

### 2.2. Method of Extraction and Preparation of EOF

After cleaning and washing, the olive, rosemary, and lavender leaves were separately subjected to hydrodistillation for 6 h using a Clevenger apparatus. The recovered oil fractions were collected separately, dried over anhydrous sodium sulfate, and stored in sealed containers at 4°C until further use [13]. Because the recovered olive leaf fraction showed a composition containing fatty acid related constituents in addition to minor volatile compounds, it is referred to throughout this study as the olive leaf derived oil fraction, whereas the rosemary and lavender fractions are referred to as rosemary leaf EO and lavender leaf EO, respectively. The test article, hereafter designated EOF, consisted of an equal volume blend of the separately prepared olive leaf derived oil fraction, rosemary leaf EO, and lavender leaf EO at a 1:1:1 (*v*/*v*/*v*) ratio.

### 2.3. Evaluation of the Phytochemicals

In accordance with the previously reported methodology [14], the individual oil fractions were chemically characterized by GC‐MS before preparation of the blend. Compound identification was based on mass spectral library matching together with retention index comparison, and relative abundances were determined from normalized chromatographic peak areas (Figure 1A–C).

**Figure 1:**
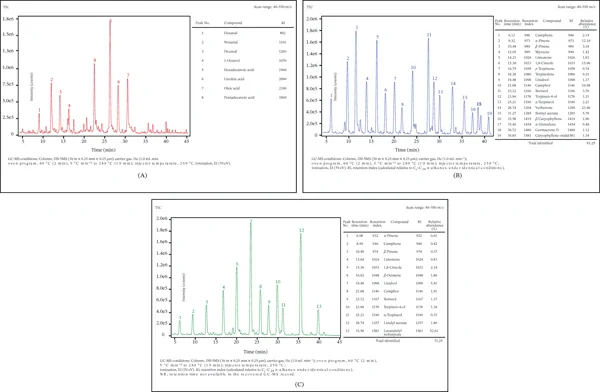
Total ion chromatograms (TICs) obtained by gas chromatography‐mass spectrometry (GC‐MS) analysis of (A) olive leaf essential oil, (B) rosemary leaf essential oil, and (C) lavender leaf essential oil. The numbered chromatographic peaks correspond to the identified constituents reported in Tables 1, 2, and 3, respectively.

### 2.4. Animals

Forty‐eight male Albino Wistar rats aged 8–9 weeks and weighing 150–175 g were obtained from the animal house of the College of Pharmacy, Al‐Kitab University, Kirkuk, Iraq (No. 16/100 on January 05, 2025). Animals were acclimatized for 1 week with free access to water and ad libitum. At the same time, they were maintained at 25^°^C ± 2^°^C on a 12‐h dark/light cycle in polystyrene plastic cages supplied with wood‐straw litter.

#### 2.4.1. Acute Toxicity Study

Animals (*n* = 18) were divided equally (*n* = 6) to three groups as low‐dose, median‐dose, and high‐dose and were starved for 3 h, then received 1 mL of a single oral dose (500, 1000, and 2000 mg/kg, respectively) of EOF that was diluted in a mixture of 2% Tween 80 (0.25 mL) and normal saline (0.25 mL). After 24 h, animals were checked for clinical adverse effects (segregation, aggression, ruffled fur, anorexia, bleeding, and diarrhea) and mortality. Finally, the NOAEL was estimated [15].

#### 2.4.2. Subchronic Toxicity Assay

Thirty animals were randomly divided into five groups of six rats each. Group I was considered the control and received tap water, whereas Groups II–V received 50, 150, 250, and 350 mg/kg of EOF, respectively. All drenching was done orally using oral gavage needle, daily for 90 days, in a volume of not more than 1 mL.

#### 2.4.3. Body Weight Measurements

Body weight was measured at different time points (Days 1, 15, 30, 45, 60, 75, and 90) for different concentrations of EOF administration.

#### 2.4.4. Animal Scarification and Sample Collection

After starvation overnight (~12 h), animals were deeply anaesthetized using xylazine (15 mg/kg) and ketamine (90 mg/kg) intraperitoneally. Blood samples were obtained (6 mL) from the caudal vena cava, of which half was collected into EDTA tubes, whereas the other half was centrifuged at 2000 RPM for 10 min to collect serum. Also, each animal had a gross necropsy to examine the external body surface, orifices, cranial, thoracic, and abdominal cavities. At the same time, excised liver and kidneys were rinsed in normal saline to remove blood and fixed in 10% formaldehyde for at least 48 h for histological analysis [16].

#### 2.4.5. Histopathological Examination and Scoring

Following fixation, the liver and kidney tissues were processed using standard histological procedures, embedded in paraffin, sectioned, and stained with hematoxylin and eosin (H&E). Histopathological examination was performed to assess the general tissue architecture and identify histopathological alterations. Liver sections were evaluated for hepatocellular degeneration, vacuolar alterations, cytoplasmic changes, and nuclear alterations. Kidney sections were evaluated for the preservation of the renal cortex and medulla, renal corpuscles (glomeruli), and tubular structures. Histopathological alterations were graded using a semiquantitative scoring system as follows: 0 = absent, 1 = mild, 2 = moderate, and 3 = severe. For each animal in the 90‐day subchronic toxicity study, two representative histological sections of the liver and two representative histological sections of the kidney were examined microscopically. The histopathological assessment was performed by a pathologist who was blinded to the treatment groups during evaluation and scoring.

#### 2.4.6. Hematological Assay

It includes determination of red blood cells (RBCs), hemoglobin (Hb), hematocrit (HCT), white blood cells (WBCs), mean corpuscular volume (MCV), mean corpuscular hemoglobin (MCH), and mean corpuscular hemoglobin concentration (MCHC) [17].

#### 2.4.7. Biochemical Assay

It includes measuring serum liver enzymes, such as alkaline phosphatase (ALP), alanine aminotransferase (ALT), and aspartate aminotransferase (AST), together with blood glucose, serum total protein, bilirubin, urea, and creatinine [17].

#### 2.4.8. Electrolytes Determination

It includes estimation of serum potassium, sodium, and chloride [17].

### 2.5. Statistical Analysis

It was performed using the Statistical Package for the Social Sciences (SPSS) (IBM, Chicago, United States, Version 27). Data from treatment groups were tested for normality using Shapiro–Wilk test. Then, the analyzed data from treatment groups were compared with that of the control group using one‐way ANOVA with Dunnett as a post hoc test. Additionally, data from the control and treated groups were compared using an unpaired *t*‐test. A *p* < 0.05 was considered significant, whereas *p* < 0.01 was set as highly significant.

## 3. Results

### 3.1. Phytochemical Analysis of the EOs

GC‐MS analysis revealed distinct chemical profiles for the olive leaf, rosemary leaf, and lavender leaf EOs. The olive leaf derived fraction showed a composition distinct from the volatile profiles obtained from rosemary and lavender. The individually reported constituents included oleic acid (6.07%), pentadecanoic acid (5.53%), hexadecanoic acid (3.86%), and linoleic acid (3.13%), together with minor volatile constituents. Because the recovered compositional record contained a substantial contribution from nonvolatile fatty acid related constituents, this fraction is referred to here as the olive leaf derived oil fraction rather than being chemically defined solely as an EO. The individually reported constituents accounted for 22.73% of the chromatographic composition (Table 1). The rosemary leaf EO exhibited a monoterpene rich profile. Verbenone (23.46%), 1,8‐cineole (15.96%), *α*‐pinene (12.10%), and camphor (10.98%) were the predominant constituents, followed by bornyl acetate (5.78%) and borneol (3.76%). The individually reported constituents accounted for 91.25% of the chromatographic composition (Table 2). The lavender leaf EO was characterized by monoterpene hydrocarbons, oxygenated monoterpenes, and other volatile constituents. Lavandulyl isobutyrate (52.61%) was the predominant constituent, followed by linalool (5.41%), 1,8‐cineole (2.14%), camphor (1.91%), *β*‐ocimene (1.86%), and linalyl acetate (1.86%). The individually reported constituents accounted for 71.25% of the chromatographic composition (Table 3). The remaining chromatographic fractions, comprising unidentified or unresolved components, accounted for 77.27% of the olive leaf–derived oil fraction, 8.75% of the rosemary leaf EO, and 28.75% of the lavender leaf EO. These components could not be confidently assigned to specific chemical identities under the applied GC‐MS conditions and were therefore reported as unidentified/unresolved components.

**Table 1:** GC‐MS composition of olive leaf essential oil.

Peak No.	Retention time (min)	Retention index	Compound	Molecular formula	Molecular weight (g mol^−1^)	Relative abundance (%)	Identification basis
1	NR	802	Hexanal	C_6_H_12_O	100.16	1.12	MS + RI
2	NR	1101	Nonanal	C_9_H_18_O	142.24	1.46	MS + RI
3	NR	1203	Decanal	C_10_H_20_O	156.27	0.84	MS + RI
4	NR	1070	1‐octanol	C_8_H_18_O	130.23	0.72	MS + RI
5	NR	1960	Hexadecanoic acid	C_16_H_32_O_2_	256.43	3.86	MS + RI
6	NR	2090	Linoleic acid	C_18_H_32_O_2_	280.45	3.13	MS + RI
7	NR	2180	Oleic acid	C_18_H_34_O_2_	282.46	6.07	MS + RI
8	NR	1860	Pentadecanoic acid	C_15_H_30_O_2_	242.40	5.53	MS + RI
			Total identified			22.73	
			Unidentified/unresolved components			77.27	Not identified

**Table 2:** GC‐MS composition of rosemary leaf essential oil.

Peak No.	Retention time (min)	Retention index	Compound	Molecular formula	Molecular weight	Relative abundance (%)	Identification basis
1	NR	932	*α*‐pinene	C_10_H_16_	136.24	12.10	MS + RI
2	NR	946	Camphene	C_10_H_16_	136.24	2.14	MS + RI
3	NR	974	*β*‐pinene	C_10_H_16_	136.24	3.18	MS + RI
4	NR	988	Myrcene	C_10_H_16_	136.24	1.42	MS + RI
5	NR	1024	Limonene	C_10_H_16_	136.24	1.83	MS + RI
6	NR	1033	1,8‐cineole	C_10_H_18_O	154.25	15.96	MS + RI
7	NR	1058	*γ*‐terpinene	C_10_H_16_	136.24	0.74	MS + RI
8	NR	1086	Terpinolene	C_10_H_16_	136.24	0.31	MS + RI
9	NR	1098	Linalool	C_10_H_18_O	154.25	1.17	MS + RI
10	NR	1146	Camphor	C10H16O	152.23	10.98	MS + RI
11	NR	1166	Borneol	C_10_H_18_O	154.25	3.76	MS + RI
12	NR	1178	Terpinen‐4‐ol	C_10_H_18_O	154.25	1.31	MS + RI
13	NR	1190	*α*‐terpineol	C_10_H_18_O	154.25	2.21	MS + RI
14	NR	1204	Verbenone	C_10_H_14_O	150.22	23.46	MS + RI
15	NR	1285	Bornyl acetate	C_12_H_20_O_2_	196.29	5.78	MS + RI
16	NR	1419	*β*‐caryophyllene	C_15_H_24_	204.36	1.96	MS + RI
17	NR	1454	*α*‐humulene	C_15_H_24_	204.36	0.48	MS + RI
18	NR	1480	Germacrene D	C_15_H_24_	204.36	1.12	MS + RI
19	NR	1581	Caryophyllene oxide	C_15_H_24_O	220.35	1.34	MS + RI
			Total identified			91.25	
			Unidentified/unresolved components			8.75	Not identified

**Table 3:** GC‐MS composition of lavender leaf essential oil.

Peak No.	Retention time (min)	Retention index	Compound	Molecular formula	Molecular weight (g mol^−1^)	Relative abundance (%)	Identification basis
1	NR	932	*α*‐pinene	C_10_H_16_	136.24	0.61	MS + RI
2	NR	946	Camphene	C_10_H_16_	136.24	0.42	MS + RI
3	NR	974	*β*‐pinene	C_10_H_16_	136.24	0.37	MS + RI
4	NR	1024	Limonene	C_10_H_16_	136.24	0.83	MS + RI
5	NR	1033	1,8‐cineole	C_10_H_18_O	154.25	2.14	MS + RI
6	NR	1048	*β*‐ocimene	C_10_H_16_	136.24	1.86	MS + RI
7	NR	1098	Linalool	C_10_H_18_O	154.25	5.41	MS + RI
8	NR	1146	Camphor	C_10_H_16_O	152.23	1.91	MS + RI
9	NR	1167	Borneol	C_10_H_18_O	154.25	1.17	MS + RI
10	NR	1178	Terpinen‐4‐ol	C_10_H_18_O	154.25	1.34	MS + RI
11	NR	1190	*α*‐terpineol	C_10_H_18_O	154.25	0.72	MS + RI
12	NR	1257	Linalyl acetate	C_12_H_20_O_2_	196.29	1.86	MS + RI
13	NR	NR	Lavandulyl isobutyrate	C_14_H_24_O_2_	224.34	52.61	MS + RI
			Total identified			71.25	
			Unidentified/unresolved components			28.75	Not identified

### 3.2. Effect of Acute Toxicity

Animals were not adversely affected by 2000 mg/kg body weight of EOF after 24 h of single oral dosing, which means that all animals tolerated the maximal EOF dosages, and the NOAEL of EOF were > 2000 mg/kg, as no adverse clinical signs or mortality were observed. The growth rate, food, and water intake of normal animals were comparable.

### 3.3. Effect of Subchronic Toxicity

#### 3.3.1. Clinical, Neurobehavioral Observations and Mortality

After 90 days of treatment, the experimental animals did not exhibit any alterations in their skin, fur, eyes, or mucous membranes, and there was no mortality among groups that received various concentrations of EOF orally. Additionally, there were no neurobehavioral observations.

#### 3.3.2. Body Weight Analysis

The body weight of rats in all categories increased gradually from day 0 to day 90 of EOF‐administration (Table 4).

**Table 4:** Mean body weight of animals at different time periods.

Groups	Body weight in gram at different time intervals (days)						
	1	15	30	45	60	75	90
I (Normal)	168.99 ± 1.34	174.41 ± 3.14	181.86 ± 2.80	186.32 ± 2.89	199.46 ± 4.56	217.81 ± 3.12	226.96 ± 1.54^∗^
II (50 mg/kg)	166.55 ± 3.54	171.57 ± 2.87	176.52 ± 2.08	181.50 ± 3.19	201.31 ± 2.87	210.01 ± 1.87	219.24 ± 2.54^∗^
III (150 mg/kg)	163.82 ± 2.65	175.78 ± 3.12	181.33 ± 2.08	189.18 ± 3.67	206.14 ± 4.12	217.65 ± 3.32	228.48 ± 3.65^∗^
IV(250 mg/kg)	153.98 ± 4.76	162.12 ± 3.90	169.26 ± 2.76	179.74 ± 3.65	209.54 ± 3.78	198.68 ± 4.22	211.01 ± 5.04^∗^
V (350 mg/kg)	163.28 ± 1.63	172.59 ± 2.04	178.80 ± 5.76	189.31 ± 4.55	209.03 ± 1.58	230.62 ± 2.65	240.88 ± 3.17^∗^

^∗^Values are significantly different at *p* < 0.05 when compared with Day 1, using ANOVA test.

#### 3.3.3. Hematological Analysis

The hematological parameters (WBC, RBC, Hb, MCV, MCH, and MCHC) were significantly decreased after administration of EOF at 150, 250, and 350 mg/kg compared with the initial day. However, these changes did not indicate any abnormal hematology when compared with normal animals (Table 5).

**Table 5:** Hematological parameter among the experimental animals.

Groups	Pretreatment							Posttreatment						
	Hb (g/L)	RBC (m/mm^3^)	WBC (Cells/mm)	HCT (%)	MCV (mm^3^)	MCH (pg)	MCH C (%)	Hb (g/L)	RBC (m/mm^3^)	WBC (Cells/m^3^)	HCT (%)	MCV (mm^3^)	MCH (pg)	MCHC (%)
I (Normal)	13.14 ± 0.3	4.94 ± 0.3	7182 ± 467	45.4 ± 3.1	87.08 ± 1.8	26.08 ± 1.3	29.68 ± 1.3	13.60 ± 0.22	5.10 ± 0.3	6056 ± 712	45 ± 2.6	88.34 ± 1.3	27.12 ± 1.4	31.28 ± 1.4
II (50 mg/kg)	13.02 ± 0.2	4.66 ± 0.3	7488 ± 856	39.2 ± 2.7	89.96 ± 1.8	29.4 ± 0.7	33.52 ± 1.4	12.30 ± 7.5	4.07 ± 1.0	5936 ± 154	37 ± 8.1	72.58 ± 18.1	23.68 ± 6.0	26.90 ± 7.0
III (150 mg/kg)	12.32 ± 0.3	4.18 ± 0.2	7466 ± 605	38.2 ± 2.1	91.2 ± 0.6	29.72 ± 0.8	32.52 ± 0.9	11.60 ± 8.7^∗^	5.20 ± 0.1^∗^	5912 ± 157^∗^	38.8 ± 8.3	71.84 ± 18.0^∗^	22.38 ± 5.6	25.02 ± 6.32^∗^
IV (250 mg/kg)	12.68 ± 0.4	4.46 ± 0.3	6874 ± 518	39 ± 3.6	88.32 ± 2.2	29.5 ± 1.7	33.32 ± 1.8	11.80 ± 6.4^∗^	4.90 ± 0.9	5142 ± 135	36.4 ± 7.9	69.9 ± 17.5^∗^	22.88 ± 5.7^∗^	26.22 ± 6.6^∗^
V (350 mg/kg)	12.34 ± 0.3	3.84 ± 0.2	7634 ± 203	37.6 ± 2.4	92.3 ± 0.7	32.2 ± 1.5	34.94 ± 1.5	12.60 ± 6.9	4.80 ± 0.7^∗^	5800 ± 143^∗^	36.7 ± 12.5	65.5 ± 11.0^∗^	26.46 ± 5.8^∗^	25.51 ± 7.5^∗^

Abbreviations: Hb, hemoglobin; HCT, hematocrit; MCH, mean corpuscular hemoglobin; MCHC, mean corpuscular hemoglobin concentration; MCV, mean corpuscular volume; RBC, red blood cell; WBC, white blood cell.

^∗^Values are significantly different at *p* < 0.05 when compared with pretreatment period, using unpaired *t*‐test.

### 3.4. Biochemical Parameters

Table 6 shows that treated group with 350 mg/kg EOF had significantly increased levels of AST and ALT, but still within the normal range. According to Table 7, only the levels of glucose and total protein were significantly increased in the 250 and 350 mg/kg EOF‐treated groups compared with the normal group. However, the values are within normal range. Moreover, Table 8 demonstrates that only the sodium levels in the 150 and 250 mg/kg EOF‐treated groups were significantly decreased compared with those in the normal group. However, the values are within normal range.

**Table 6:** Liver function test among studied animals.

Groups	AST (U/L)	ALT (U/L)	ALP (IU/L)
I (Normal)	137.99 ± 12.51	46.58 ± 2.71	25.47 ± 0.32
II (50 mg/kg)	120.71 ± 6.28	51.15 ± 3.89	23.05 ± 0.935
III (150 mg/kg)	131.4 ± 6.91	50.63 ± 6.65^∗^	22.46 ± 0.44
IV (250 mg/kg)	126.7 ± 8.65^∗^	52.39 ± 4.25	23.98 ± 0.46
V (350 mg/kg)	139.9 ± 7.41^∗^	48.26 ± 3.38^∗^	23.71 ± 0.32

Abbreviations: AST, aspartate aminotransferase; ALP, alkaline phosphatase; ALT, alanine aminotransferase.

^∗^Values are significantly different at *p* < 0.05 when compared with control group, using unpaired *t*‐test.

**Table 7:** Renal function test among studied animals.

Groups	Glucose (mg/dL)	Urea (mg/dL)	Creatinine (mg/dL)	Total Protein (g/L)	Bilirubin (mg/dL)
I (Normal)	77.75 ± 4.8	23.98 ± 2.97	0.69 ± 0.02	76.98 ± 1.15	1.77 ± 0.07
II (50 mg/kg)	84.2 ± 3.09	27.98 ± 1.61	0.73 ± 0.02	77.01 ± 1.21	1.91 ± 0.02
III (150 mg/kg)	83.94 ± 7.96^∗^	28.08 ± 2.45^∗^	0.74 ± 0.03	76.45 ± 1.79	1.92 ± 0.02
IV (250 mg/kg)	94.19 ± 4.98^∗^	25.81 ± 1.53	0.69 ± 0.01	78.02 ± 1.68^∗^	1.89 ± 0.02
V (350 mg/kg)	97 ± 5.21^∗^	26.93 ± 1.08	0.71 ± 0.02	78.18 ± 1.13^∗^	1.89 ± 0.04

^∗^Values are significantly different at *p* < 0.05 when compared with control group, using unpaired *t*‐test.

**Table 8:** Electrolyte values in serum sample from studied animals.

Groups	Sodium (meq/L)	Potassium (meq/L)	Chloride (mmol/L)
I (Normal)	159.49 ± 5.73	4.82 ± 0.23	179.2 ± 3.77
II (50 mg/kg)	133.48 ± 3.74	4.31 ± 0.13	177.3 ± 3.12
III (150 mg/kg)	127.53 ± 1.98^∗^	4.04 ± 0.09	170.27 ± 9.83
IV (250 mg/kg)	130.78 ± 2.57^∗^	4.46 ± 0.17	178.6 ± 4.91
V (350 mg/kg)	143.07 ± 5.23	4.63 ± 0.17	176.1 ± 1.63

^∗^Values are significantly different at *p* < 0.05 when compared with control group, using unpaired *t*‐test.

### 3.5. Histology of the Liver and the Kidney

After 3 months of treatment with 350 mg/kg EOF, the rats′ livers showed mild diffuse hepatocellular degeneration, vacuolar alterations with granular cytoplasm, and pyknotic nuclei. According to the semiquantitative histopathological scoring system described in the Methods, these alterations were classified as mild (Score 1). At the lower doses (50, 150, and 250 mg/kg), the hepatic cellular features were comparable to those of the control group. The histopathological scores for the liver and kidney tissues are summarized systematically in (Table 9). Kidney tissues from the treated groups showed preserved renal architecture, characterized by a distinct cortex and medulla, well‐defined renal corpuscles (glomeruli) within the cortex, and organized tubular structures within the medulla. The glomerular and tubular structures remained well defined, with no prominent structural alterations observed in the examined sections, corresponding to a histopathological score of 0 (absent). Representative histopathological findings are shown in Figure 2A–D. Additionally, a reported NOAEL values in this study are compared with other EOs (Table 10).

**Table 9:** Semiquantitative histopathological scores of liver and kidney tissues in rats following 90 days of oral administration of EOF.

Treatment group	EOF dose (mg/kg)	Liver score	Kidney score
Control	0	0 (absent)	0 (absent)
EOF‐treated	50	0 (absent)	0 (absent)
EOF‐treated	150	0 (absent)	0 (absent)
EOF‐treated	250	0 (absent)	0 (absent)
EOF‐treated	350	1 (mild)	0 (absent)

*Note:* Scoring system: 0 = absent, 1 = mild, 2 = moderate, and 3 = severe.

**Figure 2:**
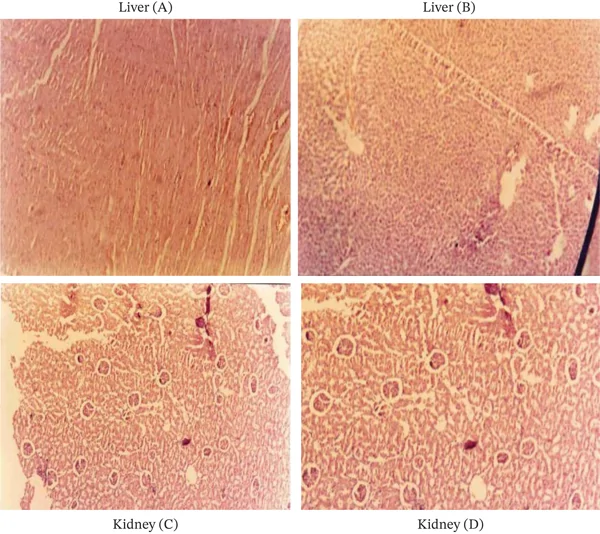
Representative histopathological findings of the liver and kidney tissues of control and EOF‐treated rats after 90 days of oral administration. (A) Normal liver architecture in control rats. (B) Liver from an EOF‐treated rat showing the observed hepatic histopathological changes. (C) Normal kidney architecture in control rats, showing a distinct cortex and medulla with well‐defined renal corpuscles (glomeruli) and tubular structures. (D) Kidney from an EOF‐treated rat showing preserved renal architecture with well‐defined glomeruli and organized tubular structures.

**Table 10:** Comparison of NOAEL values of essential oils reported in the present and previous studies.

Essential oil/formulation	Animal model	Route	Treatment duration	NOAEL	References
*Cuminum cyminum* EO	Female Wistar rats	Oral	45 days	500 mg/kg/day	[21]
Oregano EO	Wistar rats	Oral	90 days	200 mg/kg/day	[22]
*Phoenix dactylifera* seed EO	Wistar rats	Oral	14 days	< 500 mg/kg bw	[27]
*Eucalyptus globulus* EO	Mice	Oral	28 days	1000 mg/kg/day†	[28]
*Piper guineense* fruit EO	Rats	Oral	28 days + 21 day recovery	—	[29]
*Cymbopogon martini* EO	Female mice	Oral	28 days	1000 mg/kg/day	[30]
*α*‐terpineol (essential oil constituent)	Sprague–Dawley rats	Oral	90 days	146 mg/kg bw/day (males) and 500 mg/kg bw/day (females)	[31]
Garlic essential oil (GEO)	ICR mice	Oral	28 days	> 50 mg/kg bw/day	[32]
Red pine oil (*Pinus densiflora*)	Sprague–Dawley rats	Oral gavage	28 days + 14 day recovery	1000 mg/kg bw/day	[33]
EOF (olive + rosemary + lavender EOs)	Albino Wistar rats	Oral	90 days	≥ 350 mg/kg/day	Present study

## 4. Discussion

The use of herbal EOs in traditional medicine for the treatment of diseases is a common practice across many nations, especially among ancient Indian, Chinese, and Arabian peoples [18]. Several plant metabolites are commonly used for the treatment of diverse categories of diseases, such as antimicrobial, antidiarrheal, anticancer, antioxidant, anti‐inflammatory, immunomodulatory, and insecticidal activities. However, their safety profile has not yet been fully elucidated. Therefore, this study is aimed at determining the acute/subchronic toxicity profiles, along with the NOAEL, of EOF using different parameters.

Consequently, after overnight dosing with the highest concentration (2000 mg/kg) of EOF, all animals survived without showing any clinical‐neurobehavioral abnormalities. This result indicates that the NOAEL of EOF was > 2000 mg/kg body weight, as animals showed no clinical signs of toxicity or mortality at that dose. The Globally Harmonized System (GHS) classifies test compounds as Category V when they are safe at limit dose levels (up to 2000 mg/kg body weight) [19]. In this regard, a study by Bogatyrova and Naboka [20] stated that the acute toxicity of narrow‐leaved lavender extracts when administered intragastrically to white mice was classified as Class V of practically nontoxic substances. This dose is much higher than that in this study, which might be related to the fact that they used lavender leaf extract rather than an oil formulation, used different animal species, and used a single herbal oil rather than a combination of three different oil extracts. Another study reported a NOAEL for *Cuminum cyminum* EOs of 500 mg/kg/day in female Wistar rats after oral repeated dosing for 45 days, which is much lower than that observed in this study [21]. Another study by Llana‐Ruiz‐Cabello et al. [22] found the NOAEL of Oregano EO to be 200 mg/kg in Wistar rats. This disparity might be related to the duration interval, animal gender, plant species, and sample concentration.

Moreover, after 90 consecutive daily oral treatments of animals with different concentrations of EOF (repeated dosage), body weight gains and animal growth, food and water intake, abnormal clinical symptoms and gross pathological observations along with biochemical (liver and kidney function tests together with electrolytes), and histopathological (liver and kidney tissue morphology) alterations were not seen among treated animals. These outcomes confirm and approve the safe usage of EOF at different concentrations for a long period of time orally. The hematological parameters (WBC, RBC, Hb, MCV, MCH, and MCHC) were significantly decreased after administration of EOF at 150, 250, and 350 mg/kg compared with baseline data. However, these changes did not indicate any abnormal hematology (clinically not relevant) when compared with normal animals. Generally, extracted EOs are highly lipophilic that dissolve easily in fats and lipids and when they enter the bloodstream, they interact directly with the lipid bilayer of RBC membranes and compromise cell membrane permeability, leading to membrane rupture and Hb leakage.

Additionally, the increase in total protein and glucose levels following a high dose of the EOF was only marginally significant and was considered within normal limits. To our knowledge, this study is the first in Iraq to use a combination of 3 EOs in the form of EOF and to assess acute/subchronic toxicity. Thus, there is no published literature to compare the results with. However, similar patterns were detected in other studies using different herbal components, parts, formulations, and compositions, but not in studies using EOF [14, 23–28].

## 5. Limitations

The limitations of the study include lack of positive control group, single‐sex studied rats, small sample size (*n* = 6 per each group) and not including recovery group for reversibility assessment. Additionally, not studying the specific molecular biomarkers related to the toxicity of the herbal medicine in animals. Also, the toxicity to other vital organs (lungs, heart, spleen, bone marrow, and brain) was not assessed.

## 6. Conclusions

A single overnight oral dose of EOF administered to Albino Wistar rats did not result in any adverse effects at a maximum dosage of 2000 mg/kg. Consequently, different doses of EOF did not result in drastic hematological, biochemical, or histopathological changes in experimental animals up to 90 days of consecutive oral dosing. Thus, this formula is recommended for deeper testing to better characterize it, and it might then be used to treat many human diseases alongside targeted therapies in a safe manner.

## Author Contributions

A.N.A.A.: conceptualization, data collection, data analysis, study registration, and writing and revising the original manuscript.

## Funding

No funding was received for this manuscript.

## Ethics Statement

The research protocol was approved by the Ethics Committee of the College of Pharmacy, Al‐Kitab University, Kirkuk, Iraq (No. 37/88 on April 10, 2025). The study adhered to the ARRIVE (Animal Research: Reporting of In Vivo Experiments) guideline for randomization, blinding, sample size justification and OECD guideline, notably the 420‐fixed dose technique.

## Conflicts of Interest

The author declares no conflicts of interest.

## Data Availability

The data that support the findings of this study are available on request from the corresponding author. The data are not publicly available due to privacy or ethical restrictions.
